# Reduced frequency of Intravitreal methotrexate injection lowers the risk of Keratopathy in Vitreoretinal lymphoma patients

**DOI:** 10.1186/s12886-020-01464-3

**Published:** 2020-05-12

**Authors:** Xian Zhou, Xianjin Zhou, Huimin Shi, Jie Lai, Qingping Wang, Yi Li, Kun Chen, Qingjian Li, Qiang Zhou, Xia Cao, Bobin Chen, Jianjiang Xiao

**Affiliations:** 1 Department of Ophthalmology, Huashan Hospital North, Fudan University, Shanghai, China; 2 Department of Ophthalmology, Huashan Hospital, Fudan University, No.12 Wulumuqi Road, Shanghai, 200040 China; 3 Department of Laboratory Medicine, Huashan Hospital North, Fudan University, Shanghai, China; 4 Department of Hematology, Huashan Hospital, Fudan University, No.12 Wulumuqi Road, Shanghai, 200040 China

**Keywords:** Vitreoretinal lymphoma, Methotrexate, Intravitreal injection, Treatment outcomes, Keratopathy

## Abstract

**Background:**

Intravitreal methotrexate has been proven to be an effective treatment method for vitreoretinal lymphoma. However, keratopathy occurs as the major side effect during treatment in most cases. The purpose of this study is to describe the characteristics of primary central nervous system lymphoma (PCNSL) with intraocular involvement and to attempt to reduce the incidence of keratopathy caused by intravitreal methotrexate.

**Methods:**

The medical records of 22 PCNSL patients with intraocular involvement (33 eyes) were reviewed. Patients were divided into two groups. Group A (22 eyes) received the induction-consolidation-maintenance regimen, which consisted of intravitreal methotrexate injection at a dosage of 400 μg/0.1 ml twice a week for the first four weeks, weekly for the following eight weeks, and then monthly for the last nine months. Patients with a poor systemic condition were assigned to Group B (8 eyes), who were started on the treatment protocol described above and switched directly to monthly injection (9 months) when ocular remission was achieved.

**Results:**

Blurred vision (31%) and floaters (25%) were common presenting symptoms. Vitritis was the most common clinical sign and was present in 29 eyes (90%) on B-ultrasound examination. Diagnosis was made by 25G-pars plana vitrectomy, and most diagnoses were diffuse large B-cell lymphoma. Ocular remission was achieved after 8.2 (SD = 4.6) injections of methotrexate. The mean VA (visual acuity) was improved from LogMAR 0.65 to 0.3 (*P* = 0.002). Keratopathy was observed in 21 eyes (66%) after an average of 8.2 (SD = 2.3) injections. With a reduced injection frequency, the incidence of keratopathy was lowered from 86.4% (Group A) to 25.0% (Group B) without ocular recurrence during follow-up.

**Conclusions:**

Intravitreal methotrexate is a safe, effective and flexible treatment for PCNSL patients with intraocular involvement. Keratopathy is the most common adverse effect and can be controlled by reducing the injection frequency.

## Background

Vitreoretinal lymphoma (VRL), a subtype of primary central nervous system lymphoma (PCNSL), is an extremely rare type of lymphoma. In most cases, it is a non-Hodgkin diffuse large B-cell lymphoma (DLBCL) [1, 2]. Vitreoretinal lymphoma affects the vitreous body, retina and optic nerve head [1, 3]. It is estimated that 15 to 25% of PCNSL patients have concurrent vitreoretinal lymphoma [2, 4, 5]. The incidence of vitreoretinal lymphoma has been rising in recent decades as a result of the increase in the immunocompromised population, the improvement of diagnostic technology, the increase in life expectancy and the improved understanding of the disease [1, 5, 6].

The average age at diagnosis is between 50 and 60 years old, and women are more commonly affected than men at a ratio greater than 2:1 [1, 7]. Most vitreoretinal lymphoma patients have nonspecific symptoms such as blurred vision and floaters. In most cases, vitreoretinal lymphoma manifests as a “masquerade syndrome” imitating chronic uveitis. Accurate diagnosis is often delayed [8, 9]. Vitreoretinal lymphoma is initially responsive to steroid therapy because many vitreous cells are reactive lymphocytes or other inflammatory cells, which makes the diagnosis even more difficult [5, 10]. Biopsy is still the gold standard in diagnosing vitreoretinal lymphoma [4, 11]. Specimens including vitreous fluid, retina and choroid are often obtained by surgery. Diagnostic vitrectomy is the most popular method. However, due to the fragile cellularity and sparseness of vitreous specimens, the diagnosis and classification of vitreoretinal lymphoma is still challenging for ophthalmologists and pathologists [9, 12].

The optimal treatment for vitreoretinal lymphoma has not yet been determined [1, 4, 13]*.* Local treatments include ophthalmic radiation and intravitreal chemotherapeutic agents [1, 4, 9]. Intravitreal methotrexate and rituximab (anti-CD20 monoclonal antibody) are currently the main treatment methods [4, 14]. The most widely used intravitreal methotrexate treatment regimen consists of three phases: induction, consolidation and maintenance. In detail, it is comprised of injection of a dosage of 400 μg/0.1 ml twice a week for the first 4 weeks, weekly for the following 8 weeks, and then monthly for the last 9 months [15]. Keratopathy is the most common adverse effect, which ranges in severity from diffuse punctate keratopathy to severe epitheliopathy. Keratopathy is caused by a short interval between injections of intravitreal methotrexate and usually subsides when patients are treated by injection once a month. Symptoms include blurred vision, pain, tearing and photophobia, which decrease the compliance of patients. Vitreoretinal lymphoma alone has a good prognosis. However, the prognosis of central nervous system lymphoma remains poor [2, 4, 13].

Although more attention has been gradually focused on vitreoretinal lymphoma, large sample studies are rare and mostly located in Europe, the United States and Japan [2, 15]. Our study aimed to describe the characteristics of vitreoretinal lymphoma secondary to CNS lymphoma in Chinese patients and to attempt to reduce the incidence of keratopathy caused by intravitreal methotrexate.

## Methods

### Patients

We reviewed the medical records of 33 eyes of 22 consecutive PCNSL patients diagnosed with vitreoretinal lymphoma by diagnostic vitrectomy between January 2013 and January 2019 at our institution. The clinical data collected included age; gender; initial involved site; symptoms; visual acuity (VA); intraocular pressure (IOP); slit lamp examination; B-ultrasound examination; fundus photography; optical coherence tomography (OCT); diagnosis method; biopsy results; therapeutic schedule; and complications and management.

### Diagnostic techniques

All patients received pathological examination of vitreous fluid by diagnostic 25G-pars plana vitrectomy (PPV), which was performed by a skilled surgeon (Qingping Wang). Approximately 0.8 ml of undiluted vitreous fluid was aspirated by 600 cpm vitrectomy during the operation, and then 5 ml of vitreous fluid diluted with balanced salt solution was obtained. The vitreous specimens were sent to the cytopathology laboratory for cytological examinations within 30 min. Cytological examinations included smear preparation, staining with Wright’s stain, cell number counting and immunohistochemistry (CD3, CD20, PAX-5, BCL-2, and BCL-6). Interleukins (IL-10, IL-6 and IL10/IL6 ratio) were detected by enzyme-linked immunosorbent assay (ELISA).

### Treatments

Fourteen patients (22 eyes) received the induction-consolidation-maintenance regimen, which consisted of intravitreal methotrexate injection at a dosage of 400 μg/0.1 ml twice a week for the first 4 weeks, weekly for the following 8 weeks, and then monthly for the last 9 months [14, 16]. Six patients (8 eyes) were treated with an induction-maintenance regimen, and they were started on the treatment protocol and were switched directly to monthly injection (9 months) when ocular remission was achieved because the patients were too sick to tolerate high frequency treatment. The use of systemic chemotherapy and radiotherapy depended on the advice of the hematologist and the severity of the CNS lesions. Intravitreal methotrexate was not suspended unless unbearable complications occurred or the patient was too weak to receive the injection. No patients received ocular radiation. Complete clinical remission was described as the absence of obvious tumor lesions in the vitreous, retina or optic nerve head by slit lamp examination, B-ultrasound scan and OCT examination. If vitreous cells or infiltration in the retina or optic nerve head were reduced but not absent, remission was defined as partial.

### Statistical analysis

The distributions of the continuous variables were expressed as the means±standard deviations. Categorical variables are presented as percentages. The initial and final VA were compared using paired t-tests. Fisher’s exact test was used to compare differences in the incidence of keratopathy, number of patients who completed treatment and recurrence of CNS lymphoma between the two groups. The mean number of injections, cell counts of vitreous specimens, VA and follow-up time in each group were compared with t-tests. All statistical tests were 2-tailed, and a *P*-value < 0.05 was considered statistically significant. Statistical analyses were performed using SPSS 24.0 (SPSS, Inc., Chicago, IL, USA).

## Results

### Patient data

Twenty-two consecutive vitreoretinal lymphoma patients were enrolled in our study, including 12 males (54.5%) and 10 females (45.6%). The mean age at diagnosis of vitreoretinal lymphoma was 59.7 (42–73) years. Pathologic diagnoses were confirmed twice by biopsy or resection of the central nervous system. The lymphoma subtypes included DLBCL in 21 patients (31 eyes) and T-cell lymphoma in one patient (2 eyes). Eleven patients had monocular involvement, while the other 11 patients had vitreoretinal lymphoma infiltration of both eyes. Nine patients (40.9%) had CNS and eye involvement simultaneously. For the remaining 13 patients who had an initial diagnosis of PCNSL, the average time between the diagnosis of PCNSL and that of vitreoretinal lymphoma was 12.4 months. (Table 1).

**Table 1:** Demographic data, pathology, Laterality, Visual accuity

NO.	Gender	Age	Pathology	Diagnosis time (eye after CNS)	Laterality	Initial VA	Latest VA
1	M	56	DLBCL	1 month	L	0.1	0.9
2	F	68	DLBCL	2 months	R	0.8	0.6
					L	0.8	0.8
3	M	58	DLBCL	5 months	R	0.5	0.8
				1 month	L	0.5	1.0
4	M	72	DLBCL	6 months	R	0.15	0.6
					L	0.4	0.8
5	F	54	DLBCL	14 months	L	0.25	0.5
6	M	65	DLBCL	4 months	R	HM\BE	0.01
					L	0.15	FC/20 cm
7	F	45	DLBCL	23 months	R	0.2	0.8
					L	0.4	0.7
8	M	67	DLBCL	Concurrent	R	FC/20 cm	0.6
9	F	71	DLBCL	Concurrent	L	FC/20 cm	0.1
10	F	54	DLBCL	Concurrent	R	1.0	0.7
					L	0.6	0.6
11	M	56	DLBCL	32 months	R	0.4	0.8
12	M	51	DLBCL	Concurrent	L	FC/20 cm	0.5
13	M	59	DLBCL	5 months	R	0.15	1.0
					L	0.5	1.0
14	M	73	DLBCL	54 months	R	0.25	1.0
15	M	72	T cell lymphoma	2 months	R	0.6	0.8
					L	0.6	0.8
16	F	42	DLBCL	Concurrent	R	1.0	1.0
17	M	53	DLBCL	Concurrent	L	0.6	0.6
18	F	55	DLBCL	Concurrent	R	0.5	0.6
					L	0.5	0.8
19	F	65	DLBCL	Concurrent	R	0.3	0.7
					L	0.3	0.7
20	F	61	DLBCL	Concurrent	R	0.4	0.9
21	F	65	DLBCL	3 months	L	0.1	–
22	M	53	DLBCL	22 months	R	0.8	–
					L	0.8	–

*VA* Visual acuity, *M* Male, *F* Female, *R* Right, *L* Left, *DLBCL* = diffuse large B-cell lymphoma, *CNS* Central nervous system, *HM/BE* Hand movement before eye, *FC* Finger count

### Clinical features

The most common reasons that led the vitreoretinal lymphoma patients to visit an ophthalmology clinic were blurred vision (31%), floaters (25%), and decreased visual acuity with visual field disturbance (6%). However, 38% of patients diagnosed with vitreoretinal lymphoma had no ocular symptoms. The average best-corrected visual acuity (BCVA) at diagnosis was 0.22 (LogMAR 0.65). The intraocular pressures were all within the normal range.

**Fig. 1:**
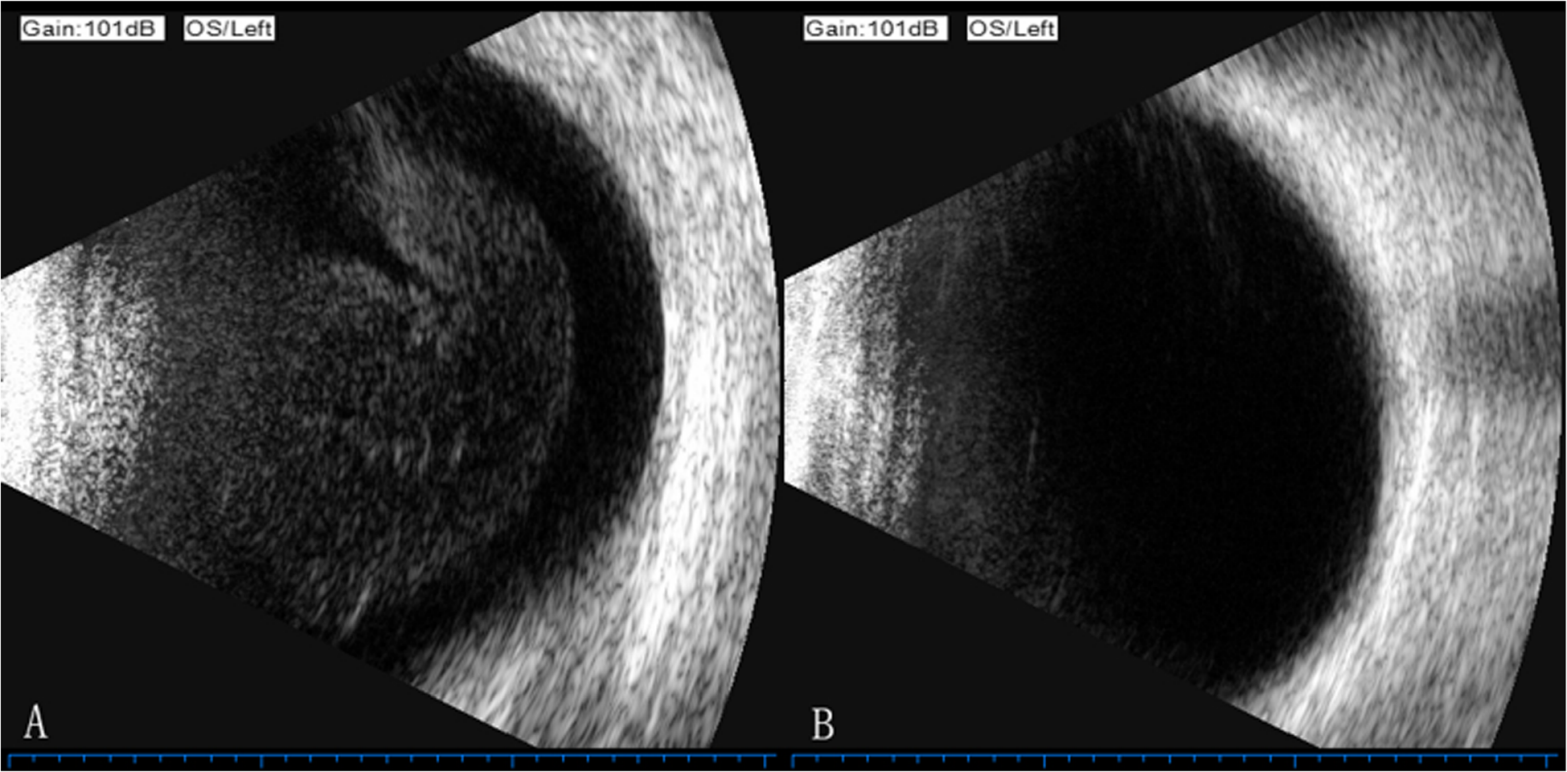
B-ultrasound scan exam of the left eye of patient 18. **a** Exam performed before the initiation of treatment with methotrexate. Note the numerous, homogeneous collections of medium-amplitude mobile echoes in the anterior and middle segments of the vitreous. **b** Exam performed after the 8th (1 month) methotrexate injection. Note the clearing o the vitreous cavity

The anterior chamber reaction was mild in all patients. Lymphoma cell infiltration in the vitreous cavity was observed in 17 eyes (53%) by slit lamp examination. To evaluate the posterior ocular segment, a B-ultrasound scan exam was much more useful. Twenty-nine eyes (90%) presented numerous, homogeneous, dispersed collections of medium-amplitude mobile echoes in the anterior and middle segments of the vitreous in the B-ultrasound scan (Fig. 1). Abnormal manifestations of the retina were recognized in 6 eyes, including hemorrhage and exudation (4 eyes), retinal atrophy (1 eye) and creamy white retinal lesions (1 eye). Fifteen eyes showed abnormalities on OCT, including outer retina fuzzy borders (10 eyes), subretinal deposits (5 eyes), retinal pigment epithelium detachment from Bruch’s membrane (4 eyes), epiretinal membrane (1 eye) and outer retina atrophy (1 eye).

### Cytological diagnosis

Cytological examination was performed to confirm the vitreoretinal lymphoma diagnosis. The biopsy samples were obtained by diagnostic 25G-pars plana vitrectomy (PPV) and sent to the laboratory within 30 min.

**Fig. 2:**
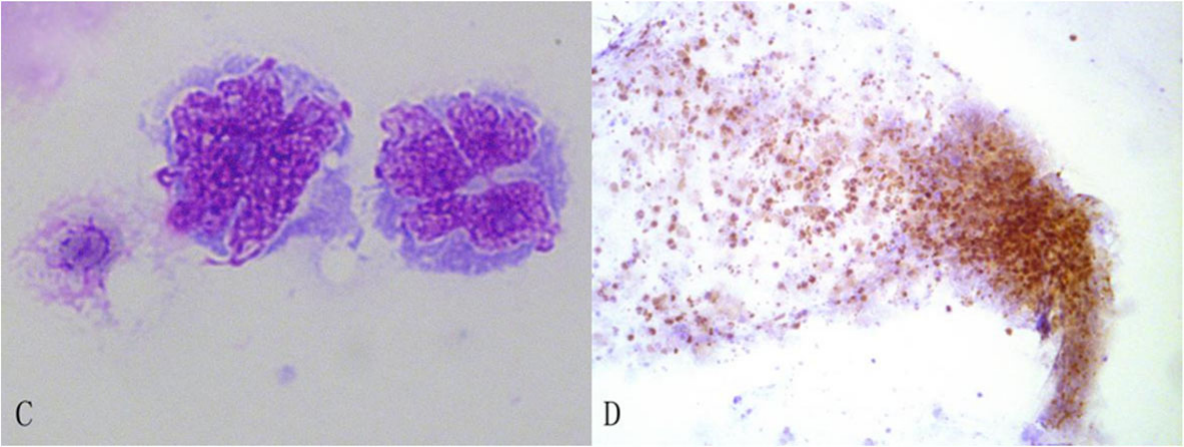
Cytological manifestations of the vitreous specimen. **c** Wright staining examination of the vitreous specimens shows the large cell size with pleomorphism and highly segmented nuclei (100 x magnification); **d** PAX-5+ B-cells were the most common infiltrating cell population (10 x magnification)

The undiluted vitreous specimens contained (121 ± 86.2) × 10^6^ cells/mL on average compared with (17 ± 21.1) × 10^6^ cells/mL in the diluted samples (*P* = 0.009). Cytological examination showed abnormal lymphocytes in 28 eyes (85%). Repeated diagnostic vitrectomy confirmed the presence of malignant lymphocytes in the remaining 5 eyes. Abnormal lymphocytes presented with a medium to large size with pleomorphism, segmented nuclei and prominent nucleoli (Fig. 2) [7, 10].

### Treatment

All patients diagnosed with vitreoretinal lymphoma received intravitreal injection of methotrexate combined with systemic therapy. The detailed treatment information is summarized in Table 2. The study population was divided into two groups according to the different treatment protocols. Group A included 14 patients (22 eyes) treated with intravitreal methotrexate according to the protocol of the induction-consolidation-maintenance regimen described above. Group B represented 6 patients (8 eyes) treated with an induction-maintenance regimen who were started on the treatment protocol and were switched directly to monthly injection (9 months) when ocular remission was achieved because the patients were too sick to tolerate high frequency treatment. In Group A, 12 eyes (54.5%) completed the induction-consolidation-maintenance regimen. Six eyes (75%) completed the induction-maintenance regimen in Group B. Twelve eyes (10 in Group A, 2 in Group B) did not complete the entire treatment but achieved ocular clinical remission. Data from 3 eyes (21, 22A, and 22B) were missing due to severe systemic complications caused by the progression of intracranial lesions. (Table 1). Thirty eyes showed a complete resolution of malignant cells in the vitreous on B scan ultrasound exam during follow-up (Fig. 1). Ocular remission was achieved after an average of 8.2 (SD = 4.6) injections (range 3–20). Nine patients (14 eyes) with recurrence of CNS lymphoma achieved ocular remission after an average of 11.2 injections (SD = 5.0). The other 11 patients (16 eyes) without CNS lesion recurrence achieved remission after 5.6 (SD = 1.7) injections (*P* = 0.001).

**Table 2:** Treatment summary

Treatment	No. of patients (eyes)
Ocular MTX + HD-MTX based regimen	10 (16)
Ocular MTX + HD-MTX based regimen + WBRT	4 (7)
Ocular MTX + HD-MTX based regimen + IT	4 (5)
Ocular MTX + HD-MTX based regimen + WBRT + IT	1 (1)
Ocular MTX + HD-MTX based regimen + APBSCT	1 (1)

*MTX* Methotrexate, *WBRT* Whole brain radiotherapy, *HD-MTX based regimen* High-dose methotrexate based regimen (systemic chemotherapy), *IT* Intrathecal chemotherapy (Dexamethasone + Cytarabine), *APBSCT* Autologous peripheral blood stem cell transplantation

The observed changes in visual acuity (VA) are summarized in Table 1. Twenty eyes showed improvement of the average VA from 0.20 (LogMAR 0.7) to 0.66 (LogMAR 0.18). Among the patients, 6 underwent cataract phacoemulsification combined with intraocular lens implantation due to age-related or complicated cataracts during the methotrexate injection course. The VA of 3 eyes decreased from 0.50 (LogMAR 0.3) to 0.16 (LogMAR 0.8). The VA of 7 eyes did not change. Paired t-tests were performed, and the difference between the initial and final VA was statistically significant (*P* = 0.001). The VA of Group A (22 eyes) improved from 0.23 (LogMAR 0.64) to 0.48 (LogMAR 0.32). The VA was improved from 0.21 (LogMAR 0.68) to 0.66 (LogMAR 0.18) in Group B. There was no significant difference in the initial and final VA between the two groups (*p* = 0.901 and *p* = 0.517, respectively).

**Fig. 3:**
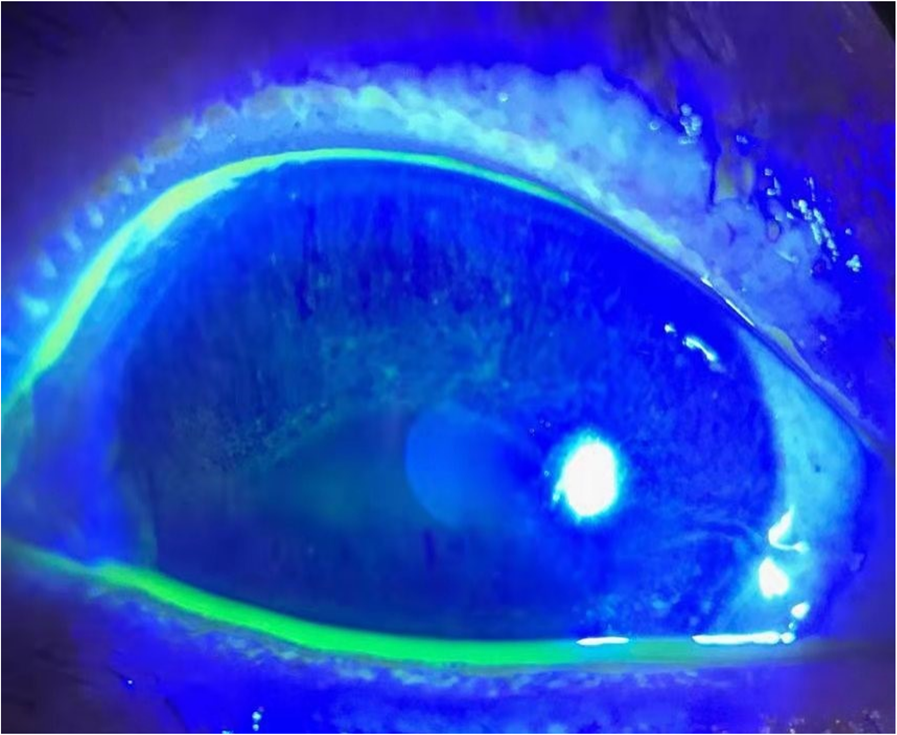
Fluorescence staining of the left eye of patient 18. Note the multiple, diffuse, fluorescent puncta, representing corneal epithelium defects

**Fig. 4:**
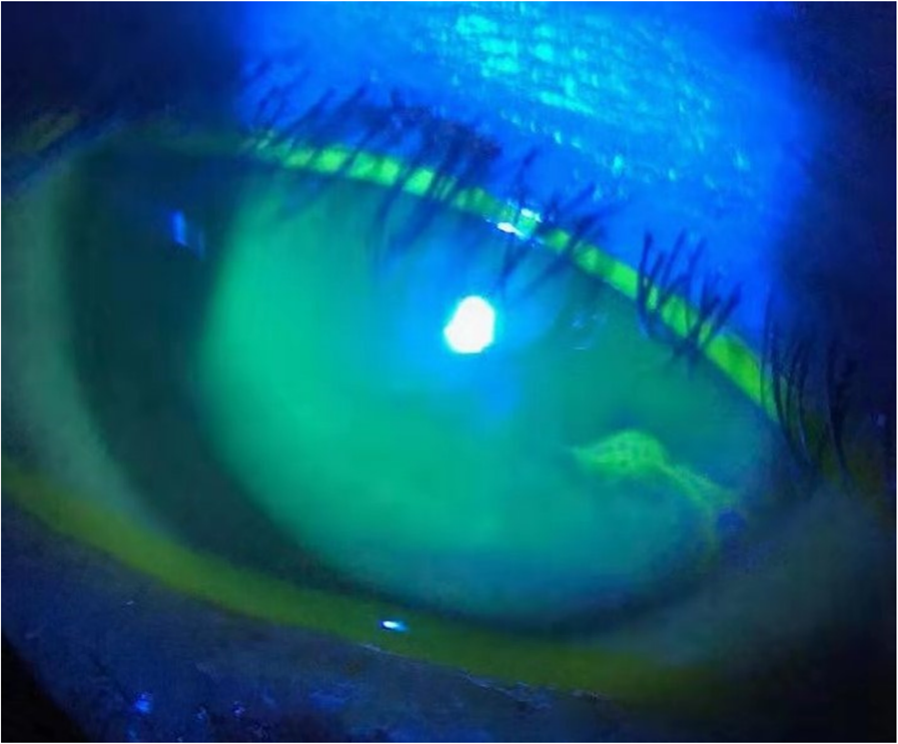
Fluorescence staining of the left eye of patient 9. Note the band-like transverse deposition of fluorescence in the infratemporal cornea

Artificial tear eye drops were used as lubricants to protect the corneal epithelium. However, keratopathy was observed in 21 eyes (70.0%) after an average of 8.2 (SD = 2.3) injections (range: 5–11). Keratopathy ranged in severity from diffuse punctate keratopathy (Fig. 3) to severe epitheliopathy (Fig. 4) and gradually subsided during the monthly injections. In Group A, 19 eyes (86.4%) exhibited keratopathy. In contrast, only 2 eyes (25.0%) developed keratopathy in Group B (*P* = 0.003). The average follow-up time from the diagnosis of vitreoretinal lymphoma was 22.6 (SD = 13.8) months, which was not significantly different between the two groups (*P* = 0.303). None of the vitreoretinal lymphoma patients had ocular recurrence during the follow-up. However, recurrence of CNS lymphoma was observed in 9 cases (6 in Group A, 3 in Group B, *P* = 0.666). The detailed data are summarized in Table 3.

**Table 3:** Treatment outcomes between Group A and Group B

	Group A (*n* = 22 eyes)	Group B (*n* = 8 eyes)	*P*-value
Keratopathy	19	2	0.003^a^
Recurrence of the CNS lymphoma	6	3	0.666^a^
Follow-up time (month)	21	27	0.303^b^
Initial VA (LogMAR)	0.64	0.68	0.901^b^
Final VA (LogMAR)	0.32	0.18	0.517^b^

Group A = treated with induction-consolidation-maintenance regimen, Group B = treated with induction-maintenance regimen. ^a^ Fisher’s exact test, ^b^ t-test. *VA* visual acuity, *CNS* central nervous system

Other complications included conjunctival hyperemia, subconjunctival hemorrhage, temporary elevation of IOP and increased lens opacity.

## Discussion

In this study, we described the clinical characteristics, diagnosis and treatment of PCNSL patients with intraocular involvement. Our study shows that the majority of patients with vitreoretinal lymphoma had an average age of 59.7 years, which is similar to that observed in other studies [1, 17].

The major symptoms observed in vitreoretinal lymphoma patients in our study were floaters, blurred vision, and decreased visual acuity. However, 38% of the patients did not complain of ocular symptoms. The proportion of symptomatic patients was relatively low. This may be because all PCNSL patients, with or without ocular symptoms, underwent ophthalmic examinations to determine the presence of vitreoretinal lymphoma. Complete and repeated ocular evaluation is suggested for PCNSL patients. Delay in assessing and diagnosing vitreoretinal lymphoma may result in a reservoir of persistent untreated disease and increase the risk of recurrence [2]. It was reported that anterior chamber reaction is often absent in slit-lamp examination [17]. The presence of vitritis is typical, especially on B scan ultrasound exam, and was present in nearly 90% of the patients in our study. Patients with numerous, homogeneous, medium-amplitude mobile echoes in the anterior or middle segment of the vitreous should be suspected to have intraocular infiltration. Multiple creamy retinal lesions [2, 9, 10], subretinal infiltrates and hemorrhagic retinal vasculitis often remit after intravitreal methotrexate injections [18].

Diagnostic vitrectomy with immediate laboratory detection, especially cytological evaluation, is critical for diagnosis [1, 9, 19]. The diagnosis of vitreoretinal lymphoma requires several approaches, including cytological examination, immunohistochemistry, flow cytometry, assessment of vitreous interleukin changes and clonality analysis [9, 20]. Most cases of vitreoretinal lymphoma are diffuse large B-cell lymphoma, and only a few cases have been reported as T-cell lymphoma, NK-T cell lymphoma or lymphoplasmacytoid lymphoma (LPL) [2, 4, 21–23]. In our study, 31 eyes (94%) were confirmed as having B-cell lymphoma by vitreous biopsy, and 2 eyes (6%) were found to have T-cell lymphoma. All the pathological types of vitreoretinal lymphoma were consistent with cerebral lesions, reflecting the common embryological origin of the eye and brain [9]. Although a variety of diagnostic methods have been developed, the diagnosis of vitreoretinal lymphoma is still challenging due to an insufficient number of vitreous specimens, poor preservation and morphological destruction of cells [1, 9, 24]. It was reported that vitreous biopsy specimens need to be sent to the laboratory for analysis quickly because lymphoma cells degrade within 60 min [12, 20]. We found that cells are more likely to remain intact and to be of higher quality when undiluted vitreous specimens are sent to the laboratory within 30 min. This also improved the diagnostic accuracy from 40%, as previously reported, to 85% in our study [25].

The optimal treatment of patients with vitreoretinal lymphoma remains undefined due to the rarity of the disease [15, 21, 26–28]. Currently, the published recommendations are from the International Primary CNS Collaborative Group (IPCG) and National Comprehensive Cancer Network (NCCN) [2, 4, 29]. It is recommended to use topical treatment, including intraocular chemotherapy or ocular radiotherapy, for eye infiltration only. A high-dose methotrexate-based regimen in conjunction with local therapy is recommended for patients with CNS involvement. For those too debilitated to receive or who have failed systemic chemotherapy, WBRT combined with ocular radiotherapy can be considered [29]. All our patients received intravitreal methotrexate therapy as recommended [14] and achieved clinical remission after an average of 8.2 methotrexate injections. Furthermore, patients with intracranial lymphoma relapse need more injections (11.2 injections) to achieve remission as a result of the increased malignancy and decreased sensitivity to methotrexate of ocular lymphoma cells [9, 30].

The improvement in VA observed in our study is similar to that observed in a previous study [14]. In our study, the VA of the two groups was preserved or improved more than expected. The early improvement of visual acuity was usually due to the clearing of vitreal haze and retinal infiltration. As the number of injections increased, visual acuity deteriorated because of keratopathy but then improved with increased injection intervals (monthly).

Keratopathy is considered a side effect related to drug toxicity [14, 18]. High doses of methotrexate inhibit the metabolism of corneal cells and the healing of the corneal epithelium, resulting in keratopathy and dry eye [18]. The lower incidence of keratopathy in Group B is probably due to the increased injection interval. As stated above, ocular remission was achieved after 8.2 (SD = 4.6) injections of methotrexate. Keratopathy was observed after an average of 8.2 (SD = 2.3) injections (range: 5–11). The number of injections needed to achieve ocular remission was similar to that needed to accumulate toxicity and result in keratopathy. This consistency probably explains why the incidence of keratopathy decreased in Group B, as patients were treated with monthly injections immediately after ocular remission was achieved.

Although the patients did not receive the complete induction-consolidation-maintenance regimen, vitreoretinal lymphoma did not recur during follow-up. There was no difference in the recurrence rate of central nervous system lymphoma between the two groups (6/14 in Group A and 3/6 in Group B). In contrast, the completion rate of treatment in Group B was 75%, which was higher than the rate of 54.5% in group A, and the patients in group B had better compliance with treatment. In consideration of the patients’ poor physical condition and the lower incidence of keratopathy, the regimen with a reduced frequency of injections is a new option for ophthalmologists. However, studies with long-term follow-up and larger samples are needed to further evaluate its safety and effect on prognosis.

Our study has some limitations. First, it is not a randomized study. In our study, the patients who were switched directly to monthly injection after ocular remission had poorer systemic conditions, which may result in bias when comparing the efficacy and adverse effects between the two groups. Second, considering the low incidence rate of PCNSL, the number of included patients in this study was relatively small. Future studies with larger sample sizes are needed to evaluate the usage of intravitreal methotrexate.

## Conclusion

In conclusion, our study describes the clinical presentation, diagnosis and treatment of vitreoretinal lymphoma patients. Our study indicates that intravitreal methotrexate is a safe, effective and flexible treatment. Keratopathy is the most frequently observed adverse effect due to the short interval between injections. Although keratopathy gradually subsides during monthly injection, it can cause discomfort and reduce compliance. A reduced frequency of injections decreases the incidence of keratopathy without ocular recurrence. Induction-maintenance regimens can be an option for ophthalmologists. However, studies with a larger sample size and long-term follow-up are needed to increase understanding of vitreoretinal lymphoma and to evaluate methotrexate therapy.

## Data Availability

The datasets used and analyzed during the current study are available from the corresponding author upon reasonable request.

## Abbreviations

PCNSL: Primary central nervous system lymphoma
SD: Standard deviation
VA: Visual acuity
LOGMAR: Logarithm of minimal angle of resolution
IOL: Vitreoretinal lymphoma
DLBCL: Diffuse large B-cell lymphoma
VRL: Vitreoretinal lymphoma
CNS: Central nervous system
IOP: Intraocular pressure
OCT: Optical coherence tomography
PPV: Pars plana vitrectomy
ELISA: Enzyme-linked immunosorbent assay
NK-T cell: Natural killer-T cell
LPL: Lymphoplasmacytoid lymphoma
